# Implementation Mapping of the Collaborative University of California Teleophthalmology Initiative (CUTI): A Qualitative Study Using the Exploration, Preparation, Implementation, and Sustainment (EPIS) Framework

**DOI:** 10.7759/cureus.64179

**Published:** 2024-07-09

**Authors:** Niloofar Radgoudarzi, Chhavi Gregg, Quinn Quackenbush, Glenn Yiu, Matthew Freeby, George Su, Sally Baxter, Christine Thorne, Rachel Willard-Grace

**Affiliations:** 1 Ophthalmology, University of California San Diego Health, San Diego, USA; 2 Informatics Services, University of California San Diego Health, San Diego, USA; 3 Family and Community Medicine, University of California San Diego Health, San Diego, USA; 4 Ophthalmology, University of California Davis Health, Sacramento, USA; 5 Endocrinology, University of California Los Angeles Health Systems, Los Angeles, USA; 6 Pulmonary and Critical Care Medicine, University of California San Francisco Health Systems, San Francisco, USA; 7 Primary Care, University of California San Diego Health, San Diego, USA; 8 Primary Care, University of California San Francisco Health Systems, San Francisco, USA

**Keywords:** implementation science research, health services research, epis framework, implementation mapping, teleretinal program, diabetic screening, telemedicine (tm), diabetic retinopathy

## Abstract

Background

This study aimed to investigate the rationale, barriers, and facilitators of teleretinal camera implementation in primary care and endocrinology clinics for diabetic retinopathy (DR) screening across University of California (UC) health systems utilizing the Exploration, Preparation, Implementation, and Sustainment (EPIS) framework.

Methodology

Institutional representatives from UC Los Angeles, San Diego, San Francisco, and Davis participated in a series of focus group meetings to elicit implementation facilitators and barriers for teleophthalmology programs within their campuses. Site representatives also completed a survey regarding their program’s performance over the calendar year 2022 in the following areas: DR screening camera sites, payment sources and coding, screening workflows including clinical, information technology (IT), reading, results, pathologic findings, and follow-up, including patient outreach for abnormal results. Focus group and survey results were mapped to the EPIS framework to gain insights into the implementation process of these programs and identify areas for optimization.

Results

Four UC campuses with 20 active camera sites screened 7,450 patients in the calendar year 2022. The average DR screening rate across the four campuses was 55%. Variations between sources of payment, turn-around time, image-grading structure, image-report characteristics, IT infrastructure, and patient outreach strategies were identified between sites. Closing gaps in IT integration between data systems, ensuring the financial sustainability of the program, and optimizing patient outreach remain primary challenges across sites and serve as good opportunities for cross-institutional learning.

Conclusions

Despite the potential for long-term cost savings and improving access to care, numerous obstacles continue to hinder the widespread implementation of teleretinal DR screening. Implementation science approaches can identify strategies for addressing these challenges and optimizing implementation.

## Introduction

Diabetes is a worldwide epidemic with multiple associated complications projected to surge in prevalence from 357 million to 783 million between 2021 and 2045 [1]. Currently, an estimated 11% of the US adult population has type 2 diabetes [2]. Diabetic retinopathy (DR) is the leading cause of blindness among working-age adults in the United States, and its occurrence is estimated to rise to 160 million cases by the year 2045 [3]. Vision loss from DR can often be prevented if it is detected and treated early, decreasing progression to blindness from 50% to 5% with appropriate treatment, making DR screening a public health priority [4]. The American Academy of Ophthalmology and the American Diabetes Association have recommended that diabetic patients undergo annual retinal examinations for DR screening [4,5]. To facilitate screening, recommendations have been incorporated into quality metrics, including the Merit-based Incentive Payment System (MIPS), a federally mandated program as part of the Medicare Access and CHIP Reauthorization Act (MACRA) that aims to incentivize quality and cost-effective healthcare [6].

Despite these recommendations, actual adherence to screening guidelines has lagged, with annual screening rates ranging from 20% to 60% [7,8]. In populations with limited access to resources, which are often at the greatest risk for vision loss due to advanced DR, these rates are even lower [9]. Major barriers to screening include socioeconomic deprivation, inadequate access to care including transportation and cost, lack of patient awareness regarding the value of regular screening, and anxiety around diabetic complications [10]. Most commonly, DR screening in the United States requires a referral and separate visit to an eye care specialist for an in-person retinal exam, presenting challenges with scheduling and traveling to another location, time off from work, barriers for insurance coverage with vision coverage being separate, and limited availability of ophthalmology appointments [7].

Teleretinal imaging where retinal images are captured at point-of-care outside ophthalmology or optometry offices and electronically transferred for interpretation by an eye care specialist has been shown to expand the capacity for retinal screening [11,12]. In recent years, DR screening image interpretation has also been successfully performed by artificial intelligence (AI) algorithms, further decreasing the need for connection to an eye care specialist for patients without disease [13]. Despite the promise of teleretinal screening, the literature lacks an in-depth exploration of teleretinal DR screening program implementation. Designing and implementing protocols that seamlessly integrate teleretinal screening programs into pre-existing clinical workflows is vital to ensure their long-term sustainability [14,15]. Liu et al. reported that system-based implementation strategies targeted at engaging key stakeholders, increasing the financial sustainability of billing models, and educating patients and providers are promising avenues to improve the performance of teleophthalmology programs [16,17].

To better understand the barriers and facilitators to the use of teleretinal imaging in primary care and endocrinology clinics, we established a Collaborative University of California (UC) Teleophthalmology Initiative (CUTI) between four UC health campuses, namely, UC San Diego (UCSD), UC San Francisco (UCSF), UC Los Angeles (UCLA), and UC Davis (UCD). The CUTI project has the following three main aims: (1) identifying barriers in teleophthalmology utilization, (2) formulating sustainable implementation practices to expand eye care access, and (3) laying the foundation for a centralized UC image repository for future retinal pathology research for advancing medical AI applications. To better understand the barriers and facilitators of teleophthalmology utilization across the UC systems, we collected baseline quantitative and qualitative data using the Exploration, Preparation, Implementation, and Sustainment (EPIS) framework to identify areas for future improvements and sustainable implementation [18]. In this paper, we will focus on our first aim, and we will dive deeper into the primary challenges and opportunities for cross-learning of teleophthalmology programs using the EPIS framework.

## Materials and methods

This study was conducted across four UC campuses: UCSD, UCSF, UCLA, and UCD. This study was approved by the UCSD Institutional Review Board as a quality improvement protocol; informed consent was waived (project #201416: Evaluating effects of teleretinal programs for improving patient care). Teleretinal project managers; primary care and specialty care physicians; and teleretinal, technology, and nursing leads were recruited to form practice innovation teams at each CUTI site to address (1) clinical workflow, (2) information technology integration, (3) financial sustainability, and (4) quality reporting. Our team also included an implementation scientist who led a series of focus groups with clinical leaders from each of the four UC campuses to perform an implementation mapping exercise [19]. During the needs assessment activity in the initial focus group, the following phases of setting up a successful teleophthalmology program were defined: camera selection, information technology (IT) infrastructure, billing, reimbursement and payment attribution, staffing, clinical workflows, training, patient education, data security and privacy, image grading, camera maintenance, and ongoing program support and sustainability. The Appendix delves deeper into the results of the focus group discussions, including needs assessment phases and considerations for each phase.

We used a mixed-methods approach to collect both qualitative and quantitative data for this study. Representatives from each campus participated in a series of two focus groups in which they walked through the phases of program implementation and identified drivers, barriers, facilitators, and lessons learned. Focus groups were recorded to supplement written notes. No compensation was provided for participation. In addition, program representatives contributed quantitative and qualitative information from the calendar year 2022 regarding the operational aspects of their programs through a standardized data collection survey.

**Figure 1:**
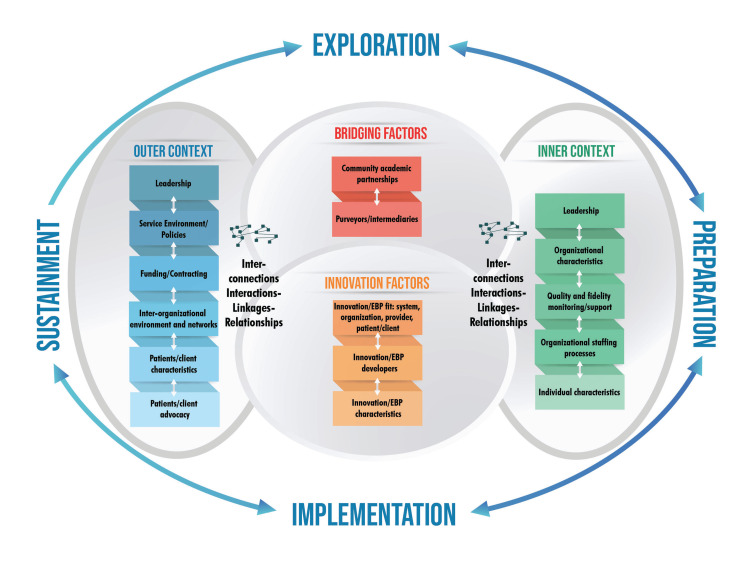
EPIS framework conceptual model. Permission to utilize the figure is granted under the Creative Commons Attribution License 4.0 [18]. EPIS = Exploration, Preparation, Implementation, and Sustainment

Qualitative data were analyzed by two team members using a reflexive thematic approach and mapped to the components of the EPIS framework [20]. The team first conducted a directed content analysis, a deductive approach in which concepts generated in the focus group were mapped to a preexisting framework (the EPIS framework shown in Figure 1). Constructs of the EPIS framework include (1) outer context, the external environment surrounding the organization; (2) inner context, characteristics within the organization; (3) bridging factors, connecting the inner and outer context; and (4) innovation factors, characteristics of the new evidence-based practices (EBPs) or innovation itself [18]. The analysis team then classified each factor identified as a facilitator or barrier and identified several recurring themes within the focus groups. Initial thematic findings were vetted with the larger study team in a meeting and revised and prioritized based on feedback.

We ran descriptive statistics of quantitative data (e.g., frequencies, means) using Microsoft Excel (Microsoft Corporation, Redmond, WA, USA). DR screening rates and image grading quality were used as quantifiable outcome measures to describe the progress of program implementation at each campus. DR screening rate in this study was defined as the number of patients screened divided by the number of active diabetic patients without bilateral enucleation aged 18 to 75. Numerator inclusion for patients screened was defined as whether a retinal exam was performed within the last 12 months for patients with known DR and 24 months for those with negative screening. Diabetic exams included patients with a record of in-person retinal or dilated eye exams by eye care professionals in the system or those reported by the patient done outside, as well as teleretinal imaging with readable images from a camera meeting DR screening guidelines from the American Optometric Association. Outside ophthalmology and optometry exams self-reported by the patients were included in our study as a surrogate for claims data, which was not available for visits outside the health systems [21].

Image grading quality was assessed on a five-point rating scale through the standardized collection survey, covering items such as image quality, consistency of image grading across graders, cross coverage for grading, consistency of offering DR screening to patients, amount of constructive feedback provided by graders for future image quality improvement. The scale ranged from 1 (poor/inconsistent) to 5 (high/consistent) for each item.

## Results

Results presented in this paper are from the calendar year 2022 and involved 20 active teleretinal sites across the four campuses, including primary care and specialty clinics. There were no mobile or community-based cameras. Across all sites, 7,450 patients were examined in the calendar year 2022. All campuses were using either 92250 or 92228 Current Procedural Terminology (CPT) codes for billing purposes, and the rates of denial for all campuses were under 20%. All four campuses reported being unsure about the financial sustainability of their programs. Across the campuses, an average of 1,565 (21%) patients were insured by Medi-Cal, 2,831 (38%) by Medicare, 2,012 (27%) by private insurance, and 1,043 (14%) had other kinds of public insurance; however, each individual campus insurance type composition varied widely based on the population they served. For example among the four campuses, the rate of Medi-Cal insured patients spanned between 1.6% and 75%. DR screening rates at each campus were as follows: UCD, 46%; UCLA, 35%; UCSD, 82%; and UCSF, 49%, for a mean of 55% across all campuses. Table 1 displays a summary of information regarding each campus’s teleretinal program.

**Table 1:** Summary information regarding teleretinal diabetic retinopathy screening programs across University of California campuses in 2022. UCSF = University of California San Francisco; UCSD = University of California San Diego; UCD = University of California Davis; FM = family medicine; IM = internal medicine

	UCD	UCLA	UCSD	UCSF
Program start date	2018	2020	2020	2012
Number of clinical sites at the time of program initiation	1	6	3	1
Number of clinical sites currently	5	6	6	3
Type of clinical office sites	4 FM/IM clinics, 1 endocrine clinic	2 FM/IM clinics, 3 endocrine clinics, 1 FM/IM/endocrine clinic	2 IM clinics, 1 FM clinic, 1 endocrine clinic	1 FM clinic, 2 FM/IM clinics
Number of eye care providers for image interpretation	4	4	3	4
Type of eye care providers	1 ophthalmologist, 3 optometrists	1 ophthalmologist, 3 optometrists	1 ophthalmologist, 2 optometrists	4 optometrists
Average turnaround time from image acquisition to interpretation	1–2 days	1–2 days	1–2 days	1–2 weeks

**Figure 2:**
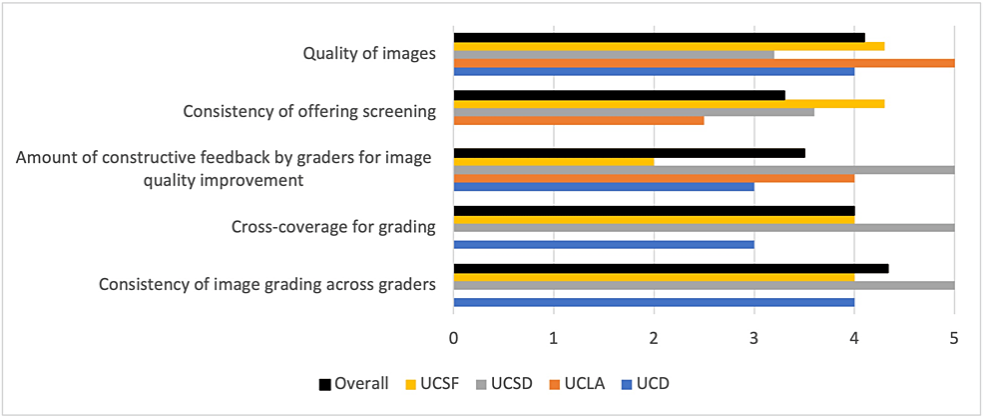
Program ratings across campuses. UCSF = University of California San Francisco; UCSD = University of California San Diego; UCD = University of California Davis

As far as image grading, all campuses opted for reporting the severity of DR in their report. Three out of four campuses also reported any noted diabetic macular edema, glaucoma, or age-related macular degeneration. Figure 2 shows program ratings for image grading quality recorded across each campus as part of the standardized collection survey and the mean rating across sites in each category. Results demonstrated the lowest reported scores for consistency of offering screening and the amount of constructive feedback by graders for image quality improvement. The highest rating was reported for the consistency of image grading across graders.

Facilitators and barriers at each phase of implementation as identified in the focus groups are described in Table 2. We organize implementation themes within the framework of the EPIS domains of outer context, inner context, innovation, and bridging factors. We also demonstrate how these domains interact [19].

**Table 2:** Facilitators and barriers in the EPIS framework identified by the program leadership during focus group meetings. Table credits: Niloofar Radgoudarzi. AI = artificial intelligence; DR = diabetic retinopathy; EPIS = Exploration, Preparation, Implementation, and Sustainment

	Facilitators	Barriers
Outer context (external environment surrounding the organization)	Incentive for meeting quality benchmarks. Attractiveness for commercial contracting	Lack of direct return on investment. Variation in coding expectations across payers
Inner context (characteristics within the organization)	Capacity expansion: better use of limited ophthalmology resources. Bulk orders increasing speed. Van-based mobile cameras improving accessibility. Standardized protocols and templates. Instant results with the use of AI platforms. Population-based outreach support	Training and quality assurance/refresher training needed. Limited staffing/high turnover at primary care offices. Limited physical space for cameras. Clinic barriers to accommodate walk-ins. Reliance on clinicians to identify/refer. Pushback from sites or staff based on the scope of perceived responsibilities
Bridging factors (the connection between the inner and outer context)	Physician and nursing champions promoting program. Incentives for increasing DR screening rates, identified as primary care priority	
Innovation factors (characteristics of the innovation itself)	Use of an ultra-wide lens increasing detection. Grading support from AI. Able to identify a range of conditions	Challenges of linking orders to correct cameras. Equipment security. Vendor support limitations. Learning curve of equipment usage. Equipment damage

Outer context. The environment outside of the implementing organization, including the policy and payment landscape, comprises the domain of the outer context within the EPIS framework. In the case of teleophthalmology, quality reporting requirements are a facilitator of the innovation, in as much as DR screening is one of the more difficult-to-attain quality indicators within commonly reported diabetes metrics. On the other hand, site representatives pointed out that there is variation in coding expectations across time and payers that pose a barrier to optimizing reimbursement for teleophthalmology. Moreover, they noted that the program requires significant initial investment and has an uncertain return on investment.

Inner context. Characteristics of the implementing organization, including the capacity and readiness for change, are part of the EPIS domain of the inner context. Key facilitators of teleophthalmology programs included expanding ophthalmology service capacity, serving more patients, and integrating eye screening into pay-for-performance and quality metrics. Common health tools such as bulk ordering, standing protocols, automated messages, and self-scheduling in the electronic health record (EHR) were beneficial. For example, the UCLA campus’s experience with using bulk ordering, in conjunction with self-scheduling and automated patient reminder alerts, showed that using these interventions simultaneously increased the odds of patients completing their eye care gap. On the other hand, limited staffing and staff turnover, space constraints, and reliance on clinician referrals were identified as barriers to the potential of the program. To create a sustainable program, low-burden workflows and continuous staff training were essential, particularly to maintain workflow quality and manage staff turnover. Sites had to consider strategies to streamline workflows to identify and outreach to patients, acquire images, and upload images for grading. Maintaining technicians’ skill levels throughout office staff turnarounds and creating viable feedback loops from the image-graders to the front-line staff were identified as recurring challenges. Staffing and training were especially challenging during the COVID-19 pandemic, with a limited number of support staff, increased patient demands, and decreased time for services.

Bridging factors. Interaction between the inner and outer context and individuals or groups spanning these two domains constitute bridging factors within the EPIS framework. Site representatives identified program champions, health system prioritization, and incentives as mechanisms that facilitated the implementation of teleophthalmology programs. The camera selection process exemplifies the interplay between these domains: affordability and leveraging payment structures were primary considerations in the outer context, while limited space in the inner context led to innovative solutions such as van-based cameras and camera-sharing. Innovation characteristics such as the use of ultra-wide lenses and systems for image upload served as facilitators to implementation. The physician and nursing champions at each clinical site played a pivotal role as the bridging factors that facilitated the implementation process and moved the EPB forward through the different phases of the framework.

Innovation. Characteristics of the technology and program comprise the innovation domain of the EPIS framework. Camera selection involved several considerations for sites, and due to the interconnectedness of the system, this decision also affects staffing and training, clinical workflow, and image grading. Affordability, space requirements, and operator training considerations were the main drivers in camera selection. Site representatives reported that the selection of the cameras was mostly based on initial investment costs, although in retrospect maintenance costs and the potential for user error resulting in ungradable image rates proved to be important factors to consider. For example, ultra-wide-field retinal imaging capabilities reduced the number of ungradable images and were strongly preferred in subsequent technology investments. Sharing a single camera between several clinics and using van-based mobile cameras were some of the solutions that clinical sites used to address the cost and space concerns.

Seamless integration of various technology systems was another major challenge in setting up a new teleophthalmology program that repeatedly came up during program leaders’ discussions. Sending the orders to the correct cameras, tracking back the ungradable images and undoing the completion of the eyecare gap, updating the primary care on the outcome of screening, and keeping track of follow-ups were some of the challenges that were addressed by using decentralized tracking systems such as internal excel sheets. Site representatives identified this as an area of significant challenge, as these systems have imperfect linkages. One of the interventions proposed by the group as a result of the implementation mapping exercise is a study to track the order and image flow through the system to detect where patients may be “lost” between systems (e.g., poor-quality images that cannot be graded and yet are registered as “complete screening” within the EHR or results that are not successfully delivered to the EHR and flagged for follow-up as needed).

One campus with the working group has implemented the use of AI in image grading. While not yet implemented at other sites, this innovation has the potential to add efficiencies to the workflow around teleophthalmology programs, as well as to expand the conditions that may be detected.

## Discussion

In this study, we used the EPIS framework to map the implementation processes of diabetic teleretinopathy screening programs across four UC campuses as part of a multi-institutional collaboration. There was considerable variation in the deployment of teleretinal screening systems, even among institutions operating under the same parent organization with the same EHR vendor. Each program at every stage of implementation was faced with a wide array of options, and decisions were made based on the program’s resources and priorities. Our goal in this study was to identify these decision points; present the wide array of choices; elucidate considerations, facilitators, and barriers; and find opportunities for cross-learning to help guide the decision-making process.

The financial sustainability of the programs was a key area of concern. Direct costs of teleophthalmology programs include expenses related to camera operator, IT integration, camera purchase, repair, maintenance, IT support, and image grading. The revenue generated from direct billing for teleophthalmology services using CPT codes often cannot entirely cover the cost of the program [22,23]. In an integrated health system, the revenue derived from teleophthalmology programs stems from four areas, namely, revenue directly generated from the telemedicine service, future revenue stemming from patient referrals, payments from incentive programs, and cost savings achieved from replacing in-person clinic exams [22]. The UCD program published a cost analysis study showcasing a reduction of $42.53 per patient when considering both direct and indirect ways of revenue generation by teleophthalmology programs, which was in line with the number reported by similar studies [22,23]. Despite teleophthalmology programs’ shortcomings in creating directly generated revenue, ensuring their financial feasibility is crucial to reap their long-term financial benefits and improvements in quality metrics. It is noteworthy that all four campuses participating in this study report uncertainty around the financial stability of their programs. The main challenges in this area remain the intricate nature of the billing environment, including variations in the approval of different billing codes, frequent regulatory changes in this area including changing definitions of CPT codes, and changes in reimbursement amount by code. Currently, teleretinal CPT codes 92227 and 92228, which code for imaging of the retina for the detection or monitoring of disease with remote clinical staff review and report, fall under Ambulatory Payment Category 5732 (Level 2 Minor Procedure) and their reimbursement rate is around $33. CPT 92250 covering fundus photography in the eye clinic by a physician or ophthalmology technician is categorized as APC 5734 (Level 4 Minor Procedure) and reimburses around $55. The newer CPT code 92229 which allows for autonomous AI detection and monitoring of DR is considered under APC 5733 (Level 3 Minor Procedures) and results in reimbursement around the $55 range, billed higher than reports generated by human readers [6,24,25].

Health IT considerations comprised another area of opportunity for cross-institutional learning. Proper integration of the current EHR system and the picture and archiving communication system (PACS) is necessary to transfer of orders, images, and reports between relevant parties, across multiple departments. This is a notable challenge in ophthalmology as ophthalmic image vendors utilize proprietary platforms with low rates of information standards adoption [26]. Setting up IT infrastructure for split billing between departments, linking the orders to the correct cameras, routing through PACS systems, building reading queues, reversing the completion of the eye care gap in case of ungradable images, sending the outcome report to the ordering physician, prohibitions against self-referral by readers to their own department requiring follow-up to be ordered by the imaging practitioner, formulating patients’ next steps based on screening results, and keeping track of follow-ups were some of the challenges in the health IT realm. Some of these challenges were addressed by decentralized tracking systems such as internal spreadsheets, which although crucial in maintaining accuracy within the limits of the current infrastructure, can increase the work burden due to being less automated. Navigating these challenges can be complicated and creating a process flow map, consisting of the decision points in setting up the IT build for a new teleophthalmology program can be a valuable resource and an area for a potential complementary study. One important consideration while designing integrated IT systems is ensuring the lightest possible additions to workflow to improve usability and avoid unnecessary burden and burnout [27]. One way to do so is taking advantage of automated elements in the system and one potential solution is the use of AI in image grading, report, and referral to increase automation. Although IDx-DR was the first Food and Drug Administration-cleared autonomous AI diagnostic system with image interpretation abilities, there remain substantial challenges in the real-life use of AI systems in this area, another opportunity for further investigation [28].

One area of significant variation among programs was on patient outreach avenues. In the current literature, it has been reported that postal and telephone reminders and having pre-scheduled screening appointments instead of open appointments are effective strategies for increasing participation in screening programs. Interventions aimed at simplifying screening focus on ways to facilitate scheduling appointments such as offering tests at routine consultations and sending invitations with pre-scheduled appointments [29,30]. UCLA’s positive experience with the use of bulk ordering in conjunction with automated patient alerts and self-scheduling is an example of a successful combination of outreach efforts. Using the resources available at each institution to generate innovative outreach strategies targeting individuals and communities can increase patient participation in screening programs. Following the implementation of these outreach strategies, another area deserving further exploration is developing a system to track further patient follow-up beyond mere scheduling of the screening appointment.

A unique aspect of our teleophthalmology initiative was the fact that we started this process with an implementation science framework in mind. The majority of health promotion services fail to systematically plan the adoption and implementation strategies to deliver an intervention. This limits the reproducibility of these interventions and hinders the potential to re-apply their findings in other settings [19]. We used steps of implementation mapping to facilitate strategic preparation for the dissemination and execution of our program by considering goals, needs, facilitators, barriers, and methods essential for success. We also synthesized our findings onto the EPIS framework, which enabled us to look at our intervention in its environmental context and helped elucidate the interconnectedness of different phases of implementing an EBP. This serves as a real-life example exercise of closing the gap between theory to practice in the field of implementation science.

Our study had some limitations. It mainly relied on data gathered from institutional leaders. More comprehensive and larger-scale studies from a more diverse group of members of the medical community are needed to investigate the advantages and obstacles of teleophthalmology programs. Second, it would be beneficial to define more quantifiable measures of program success in different areas to keep track of the program’s course over time and be able to find associations between each intervention and program outcome. Longer-term studies are needed to delineate these relationships and determine the overall impact of the program, for example, as far as visual outcomes of patients screened or patient and provider satisfaction.

## Conclusions

The use of telemedicine to expand access to DR screening is a promising approach to enhancing access to care and meeting quality standards goals. Understanding and addressing a myriad of implementation challenges for telemedicine DR screening programs is crucial to enable this innovation to be brought to scale. Our study uses a well-known implementation framework called EPIS to highlight variations, considerations, barriers, and facilitators in the process of planning and deploying our UC teleophthalmology initiative across four UC campuses. More formalized mapping of each decision point, consideration factors, and long-term outcomes are necessary for developing a guideline for future new teleretinal programs to reference. These types of implementation science studies will be paramount to understanding healthcare systems and developing system-level solutions for the long-term sustainability of teleophthalmology programs and improving screening rates for vision-threatening diseases.

## Appendices

**Table 3:** Needs assessment phases and subcategories identified based on focus group implementation mapping exercise between clinical leaders of our teleophthalmology programs. Table credits: Niloofar Radgoudarzi. AI = artificial intelligence; CPT = Current Procedural Terminology; EHR = electronic health record; PACS = picture and archiving communication system

	Considerations
Camera selection	Cost: Wide-field vs. conventional camera, hand-held vs. countertop. Technical expertise required, e.g., dilation requirement. User-friendliness vs. error-proneness. Long-term maintenance costs, e.g., subscription fees and vendor support
IT infrastructure	Image interpretation workflow (linking image capture and interpretation documentation systems, e.g., PACS and Epic). Automatic update of health maintenance tracking system. Split billing between departments. Sending screening results back to the referring doctor. Guiding the patient’s next steps based on screening results, e.g., sending to ophthalmologist vs. picking a date for next screening. Workaround for next steps after poor-quality imaging reported. Tracking follow-up. Data sharing privacy concerns regarding building a repository
Billing	Variations in billing codes approval by insurance (92227, 92228, 92225) and frequent regulatory changes. Rates of denial. Modification of the CPT code depending on the interpretation
Staffing and training	Limited staffing at non-ophthalmology offices. Scope of work and perceived responsibilities of staff. Need for physician and nursing clinical champions to promote the program. Need for dedicated time for ongoing training for new staff and floating staff as well as scheduling refreshers. Considering unique learning curve for each camera. Requirement for on-the-job training geared toward each device. Making tip sheets for quick referencing. Defining metrics for tracking the quality of images
Clinic workflow	Ways of reducing physician dependency of system by different ways of automated ordering like bulk ordering and self-scheduling. Workflow for archiving images. Need for protected time for accommodating walk-ins. Physical space for cameras. Instant results using AI platforms. Unique aspects of using van-based mobile cameras. Standardizing protocol and ensuring the lightest workflow possible. Expansion of services, and considerations for the number of cameras and sites
Image grading	Granularity of grading system, e.g., choice of looking for other pathologies. Possibility of using AI and determining human overread with AI. Defining image grading requirements for billing compliance and training purposes. Choosing image graders, ophthalmologists vs. optometrists
Patient communication and outreach	Patient education about the importance of screening and methods of scheduling. Bulk ordering. Self-scheduling options for patients. Avenues for patient contact, e.g., My chart messages. Identifying care gaps accurately on EHR. Ways of sustaining referral rates, e.g., incentives, primary care engagement, clinician, and staff education
